# Effect of tissue microenvironment on fibrous capsule formation to biomaterial-coated implants

**DOI:** 10.1016/j.biomaterials.2021.120806

**Published:** 2021-06

**Authors:** Jamie L. Hernandez, Jaehyung Park, Shan Yao, Anna K. Blakney, Hienschi V. Nguyen, Bob H. Katz, Jeffrey T. Jensen, Kim A. Woodrow

**Affiliations:** a Department of Bioengineering, University of Washington, 3720 15th Ave NE, Seattle, WA, 98105, USA; b Oregon National Primate Research Center, Oregon Health & Science University, 505 NW 185th Ave, Beaverton, OR, 97006, USA; c ContraMed LLC, 900 E. Hamilton Ave, Campbell, CA, 95008, USA

**Keywords:** Electrospun fibers, Foreign body reaction, Female reproductive tract, Intrauterine device, Immune privilege, Sclerosing agents

## Abstract

Within tissue exposed to the systemic immune system, lymphocytes and fibroblasts act against biomaterials via the development of a fibrous capsule, known as the foreign body reaction (FBR). Inspired by the natural tolerance that the uterine cavity has to foreign bodies, our study explores the role of microenvironment across classical (subcutaneous) and immune privileged (uterine) tissues in the development of the FBR. As a model biomaterial, we used electrospun fibers loaded with sclerosing agents to provoke scar tissue growth. Additionally, we integrated these materials onto an intrauterine device as a platform for intrauterine biomaterial studies. Polyester materials *in vitro* achieved drug release up to 10 days, greater pro-inflammatory and pro-healing cytokine expression, and the addition of gelatin enabled greater fibroblast attachment. We observed the materials that induced the greatest FBR in the mouse, had no effect when inserted at the utero-tubal junction of non-human primates. These results suggest that the FBR varies across different tissue microenvironments, and a dampened fibrotic response exists in the uterine cavity, possibly due to immune privilege. Further study of immune privileged tissue factors on biomaterials could broaden our understanding of the FBR and inform new methods for achieving biocompatibility *in vivo*.

## Highlights

• Biomaterials examined for FBR across subcutaneous and uterine environments.
• Polyester fibers can achieve physicochemically diverse drug release up to 10 days.
• Polyester fibers induce higher pro-inflammatory and pro-healing cytokine expression.
• PLGA/PCL/Gel/SN initiated the greatest FBR subcutaneously in mice.
• Targeted fiber/IUD did not initiate scar tissue growth in a uterine NHP model.

## Graphical abstract

**Figure:**
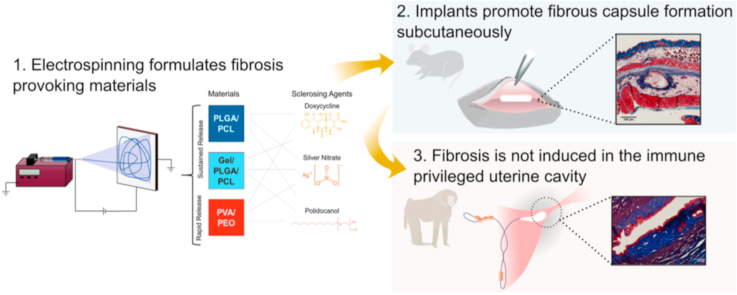

## 1 Introduction

The foreign body reaction (FBR) describes the host response towards biomaterial implants [[1], [2], [3], [4]]. The FBR begins with the spontaneous absorption of blood plasma proteins onto the biomaterial surface, forming a provisional matrix for FBR relevant cell types [1,[4], [5], [6], [7]]. Although neutrophils are the first immune cells active in the FBR, macrophages are the primary arbiters of this response [1,3,4]. Polarized macrophages direct the local tissue response towards either a state of chronic inflammation or tissue remodeling [1,3], which is further shaped by various cytokines that trigger migration of immune cells and fibroblasts. Fibroblasts aid in wound healing and are also responsible for the deposition of collagen-rich scar tissue known as the fibrous capsule [1,3,4] This pathology of the FBR is well characterized from the perspective of various biomaterial properties, but is less studied across different tissue microenvironments such as the local cellular, extracellular, chemical, and tissue mechanical properties at homeostasis [1,2,[8], [9], [10], [11], [12]].

Biomaterial properties have been extensively investigated for their contributing role in the FBR. Hydrophobic materials show greater protein adsorption and macrophage adhesion, which may promote fibrotic encapsulation [1,5,8,[13], [14], [15]]. For hydrophilic materials, macrophages have shown greater adhesion to cationic as compared to anionic or nonionic implants [1,14]. Material topography has also been shown to impact development of the FBR, with smooth and flat implants inducing greater foreign body giant cell (FBGC) adhesion and fibrotic capsule formation [1,16]. Mechanical mismatch between biomaterial and the local tissue has also been shown to provoke pro-inflammatory responses [1,17,18]. As such, strategies to prevent the FBR have focused on changing attributes of the biomaterial itself such as employing inert polymers, surface modifications, control of geometry, and patterning techniques to improve biocompatibility and tissue integration [1,3,5,8,19]. Although the FBR is well studied from the biomaterials engineering perspective, less is known about the role of different tissue microenvironments [8,10,12,15]. Sites of immune privilege are particularly interesting to consider given their immunosuppressive bias that is distinguished by large populations of regulatory T-cells [20], increased expression of anti-inflammatory cytokines [21] and matrix metalloproteinases (MMPs) [22,23]. Whether immune privilege alone can hinder development of the FBR induced by biomaterials has not been investigated, and could have important implications for future design of biomaterial implants.

The FBR has been best characterized within the subcutis due to the testability in mice and the minimal invasiveness of the procedure. While these factors have made the subcutaneous implant model the standard for assessing implant biocompatibility, it has narrowed our understanding of microenvironmental factors that could be exploited for controlling the FBR [2,15,[24], [25], [26], [27]]. In contrast to subcutaneous tissue, specific regions of the body exist that confer immune privilege in order to protect tissue function, including the eye, brain, and reproductive organs [28]. Immune privilege in the uterus prevents host recognition of haploid germ cells, paternal antigens in sperm cells, as well as embryos and fetuses [[28], [29], [30]]. Despite this privileged immune status, some biomaterial foreign bodies are known to provoke an immune response in the uterus. In fact, the contraceptive mechanism of intrauterine devices (IUDs) is due in part to inflammation generated towards a foreign body [[31], [32], [33], [34]]. Although IUDs provoke an immune response, these devices are not subject to fibrous capsule formation and can be readily removed [33]. Intrauterine fibrosis is known to arise only under chronic provocation. For example, repeated exposure to *Chlamydia trachomatis* can cause chronic inflammation of the fallopian tube that leads to tubal occlusion and infertility [35,36]. Cytotoxic agents that cause sclerosis have also been administered within the uterine cavity as a method to induce scar tissue growth for permanent contraception [[36], [37], [38], [39], [40]]. The uterus is an interesting compartment to investigate microenvironmental effects on the FBR because it is a highly vascularized organ but, as described above and in contrast to the subcutis, shows differential responses to foreign bodies. Investigating microenvironmental effects in the uterus towards biomaterials previously described in the subcutis as either inert or capable of generating an inflammatory response could demonstrate a larger role for leveraging host factors to inhibit the FBR to biomaterial implants [1,41,42].

Here, we investigated the relationship between the FBR resulting in fibrous capsule formation across the classical (subcutaneous) and immune privileged (uterine) environments. Biomaterials eluting various sclerosing agents were used to further provoke a FBR towards implants. Electrospinning was implemented as the material fabrication method for its known ability to efficiently encapsulate physicochemically diverse agents, modulate drug release rates [[43], [44], [45], [46], [47]] and for ease of integration onto an IUD. Materials were screened for a pro-fibrotic response both *in vitro* and in a subcutaneous mouse model, then highly fibrotic materials were placed via an IUD into an intrauterine baboon model. Although the biomaterials proved to be highly fibrotic in the subcutis, the same materials did not provoke a response within the uterine cavity. This is the first study to our knowledge showing that uterine immune privilege alone may hinder development of a fibrotic FBR. The foreign body response to biomaterials has been investigated primarily from the perspective of biomaterial attributes (e.g., surface chemistry, topology, mechanical, etc.), but only minor attention has been given to the role of host microenvironmental factors. We show that using biomaterials with a record of inducing a strong foreign body response, and even when combined with fibrotic inducers, fail to initiate fibrosis when transplanted into a site of immune privilege in the uterus. Our results suggest the importance of identifying host factors that could be leveraged in combination with biomaterials engineering to reduce the foreign body response to medical implants in non-immune privileged tissue microenvironments.

## 2 Materials and methods

### 2.1 Electrospinning fiber formulations

Poly(lactic-co-glycolic acid) (PLGA, 50:50 L:G, acid terminated, inherent viscosity = 0.55–0.75 dL/g, Lactel Absorbable Polymers) and polycaprolactone (PCL, Mw = 80,000, Sigma-Aldrich) were blended at a ratio of 80:20 (wt./wt.) in 15% (wt./v) hexafluoroisopropanol (HFIP, Oakwood Chemical). PLGA/PCL/Gelatin (Gel, Type A from porcine skin, Sigma-Aldrich) was created at a ratio of 64:16:20 (wt./wt./wt.). Sclerosing agents were added into the aqueous polymer solution at defined weight percent loadings in respect to polymer mass. The tested sclerosing agents include doxycycline hyclate (Dox, MP Biochemicals), nonaethylene glycol monododecyl ether for polidocanol (PD, Sigma-Aldrich), and silver nitrate (SN, Sigma-Aldrich). Polymers were needle electrospun using a 1 mL glass syringe and 21 G, 1.5-inch needle. A voltage of 10–13 kV was applied, and polymer was extruded at a rate of 40 μL/min. Poly(vinyl alcohol) (PVA, 87–90% hydrolyzed, MW = 30,000–70,000, Sigma-Aldrich) and poly(ethylene oxide) (PEO, Mw = 400,000, Scientific Polymer Products, Inc.) blends were added at a ratio of 86:14 (wt./wt.) into 17.4% (wt./v) deionized water with 0.0224% (wt./v) sodium chloride. PVA/PEO materials were electrospun using an Elmarco Nanospider (Liberec, Czech Republic). The carriage rate was set to 350 mm/s, with an electrode distance of 200 cm, and with 100 kV voltage difference. Electrospun fibers were prepared into 10 mg, 1.5 cm long sections, UV sterilized for 30 min per side, and hand-rolled cylinders secured using 20% (wt./v) PVA/water glue.

### 2.2 Sclerosing agent release, encapsulation, and measurement

Release studies were conducted using the drug loading implemented for mouse studies and at sink conditions. Dox and PD used 1x DPBS (Mediatech, Inc.) as the release medium, and ultrapure water (Millipore Sigma) was used for SN. Release media samples were taken at specified timepoints starting at 5 min and out to two weeks. Percent release was quantified as a ratio of the measured sample signal (x_Sample_) to the signal of full theoretical loading (x_Theo_), with signal from the blank release media (x_Blank_) subtracted out, as shown in the equation below:

$$\%\;release=100\times \frac{x_{Sample}-x_{Blank}}{x_{Theo}-x_{Blank}}$$

Encapsulation efficiency of drug within the materials was measured after dissolving polyester fibers in dimethyl sulfoxide (DMSO, BDH/VWR Analytical) and PVA/PEO fibers in water. Percent encapsulation (% EE) was calculated using the equation below:

$$\%\;EE=100\times \frac{Measured\;drug\;mass\;in\;fiber\;sample\;}{Theoretical\;drug\;mass\;in\;fiber}$$

Dox was quantified by high-performance liquid chromatography (HPLC) (Simadzu Prominence) using a method referenced from Kogawa et al. (2013) [48]. Briefly, we used a detector wavelength of 360 nm, injection volume of 20 μL, and A C18 column (Phenomenex Kinetex) for the stationary phase. The mobile phase 75:25 (v/v) water/ACN (HPLC grade, Fisher) with 0.1% (v/v) trifluoroacetic acid (TFA, Sigma-Aldrich) was used, resulting in a retention time of 10 min for Dox. SN release was quantified using inductively coupled plasma optical emission spectrometry (ICP-OES) (PerkinElmer Optima 8300). Silver was detected using the preferred emission wavelength of 328.068 nm. PD approximation by thin-layer chromatography (TLC) was adapted from Hahn-Dienstrop (2007) [49]. Samples, drug spiked positive control samples, blank fiber negative control samples, and PD standards were all spotted by glass capillary onto a single TLC plate (250 μm, particle size 10–12 μm, Millipore Sigma). The plate was dried (approximately 1 min), then saturated in Dragendorff's reagent (Sigma Life Science) for 1 min, removed, and set flat to dry again. Once the color had developed, images of the plates were collected using a standard office scanner (Aficio MP 301, Ricoh, USA). Plate images were analyzed using ImageJ (FIJI/ImageJ 1.51s, National Institutes of Health, USA) [50] software to quantify spot pixel intensity in the blue channel.

### 2.3 Cell culture assessment of cytokine expression and cell attachment

To assess the induction of inflammatory responses, RAW 264.7 macrophages were used as the cell model, (kindly gifted by Dr. James Bryers). Cells were cultured in Dulbecco's Modified Eagle Medium (DMEM, 4.5 g/L d-Glucose, l-glutamine, 110 mg/L sodium pyruvate, Gibco) supplemented with 10% (v/v) fetal bovine serum (FBS, Gibco) and 1% (v/v) penicillin-streptomycin (P/S, Gibco) and seeded 0.5 × 10^6^ per well in a 6-well plate. Drug treated cells were cultured with 10 mg of fiber, or drug spiked media at the equivalent dose. For a positive control, 100 ng/mL of lipopolysaccharide (LPS) was added to the media. Media was collected after 48 h of culture, centrifuged at 1500 RPM for 10 min, and the supernatant was stored at −80 °C until analysis. Expression of TNF-α, IL-1β, and IL-10 were determined via enzyme-linked immunosorbent assay (ELISA) kits purchased from PeproTech and used as instructed.

To test fibroblast attachment, fiber mats were controlled for surface area and mass, and delivered onto glass coverslips with a final fiber mass of 10 ± 0.1 mg. Collagen coated coverslips were used as a control for cell attachment, and were created with 40 μL collagen (rat tail collagen type I, Corning) diluted to 50 μg/mL in 0.2 N acetic acid (Fisher Scientific). Cultured NIH 3T3 fibroblasts were resuspended in serum free, high-glucose DMEM with 1% P/S. Coverslips in a 6-well plate were seeded with 2.5 × 10^5^ cells. After 24-h of culture, the quantity of attached cells on the material was measured using Cell Titer Blue (Promega, used as instructed).

### 2.4 Subcutaneous implant surgeries

Murine research was approved by the Institutional Animal Care and Use Committee (IACUC) at the University of Washington, and all guidelines for care were followed. Implantation studies were conducted using 8 to 12-week-old female C57BL/6J mice (Jackson Laboratory). Mice were anesthetized using isoflurane delivered by a precision gas vaporizer and given a subcutaneous injection of buprenorphine hydrochloride (0.05 mg/kg) as an analgesic. Two incisions were made just off the midline of the dorsum. A single pocket was made from each incision in the subcutaneous space above each scapula using blunt dissection. One fiber/drug implant was placed in each pocket. After 28 days, entire pocket sections of the mouse were excised and fixed in a formalin solution made from 4% (v/v) paraformaldehyde (Electron Microscopy Sciences, 40% aqueous solution). Tissues were sectioned and stained with Masson's trichrome. Images were captured using a Sakura VisionTek Digital Microscope.

### 2.5 Fiber IUD placement in baboons

**Fig. 1:**
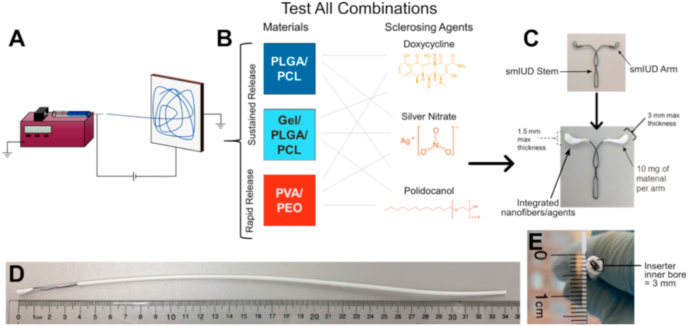
Material design and integration onto an IUD. (A) Schematic of the electrospinning process, and (B) the material drug combinations tested throughout this study. (C) Image of the modified VeraCept® nitinol wire IUD frame (top) and the proposed combined IUD/fiber device with required dimensions for the wrapped electrospun fibers (bottom). Fiber dimensions were constrained by the IUD insertion tube required for placement of the (D) folded fiber-IUD. The (E) inner bore of the inserter is 3 mm in diameter.

Experiments conducted with baboons were approved by the IACUC at Oregon Health and Sciences University. We created drug-eluting fiber intrauterine devices (IUD) by integrating 10 mg of electrospun fiber materials onto each arm of a nitinol wire IUD frame (Fig. 1C). The nitinol wire frames were supplied by ContraMed LLC, (Campbell, CA). Three healthy adult female baboons received IUDs; blank-fiber (PLGA/PCL/Gel) device (n = 1, *Papio hamadryas*), and silver nitrate loaded fiber devices (loaded at 60% (wt./wt.) SN in PLGA/PCL/Gel Fibers) (n = 2, one each *P. hamadryas*, P. Anubis). The females received general anesthesia and underwent transcervical placement of the IUDs using an insertion tube under ultrasound guidance. Successful placement of the devices was confirmed by contrast hysterosalpingogram using fluoroscopy. After 28-days, the animals underwent humane euthanasia and necropsy. A gross dissection of the extirpated reproductive tract was performed, with the uterine cavity opened to evaluate the position of the IUD arms in the cornual region. Tissue sections taken from this region, the intramural fallopian tube, the endometrium, and tubal isthmus were collected for histological analysis. These tissues were paraffin embedded, and representative sections stained with hematoxylin and eosin, and with Masson's trichrome.

### 2.6 Image analysis of histology to measure fibrotic response to study materials

Supplementary Fig. 5 illustrates our process for the quantitative image analysis of the murine subcutaneous histology. In the subcutis, we focused on measuring inflammation and collagen as correlates of the FBR. We first defined three regions of interest (ROI) to perform unbiased measurements. The first ROI is defined by a line, intersecting the center point of the implant in the image of the implant cross-section, and drawn perpendicular to the skin surface (L1, solid white line). To approximate the implant location for materials dissolved by the study endpoint (PVA/PEO), the center ROI was determined to be at the center point of observed collagen or immune cell staining. Two additional ROIs are defined as parallel lines (solid white line) 1 mm to the left (L2) and right (L3) of L1. ImageJ was used to take three measurements at each of these ROIs: (1) subcutis length = length between the two muscle layers (dashed white line, Lsc), (2) inflammation = total length of accumulated immune cells stained within the subcutis, marked by red stain and additionally identified by cell morphology (dashed black line with in-turned black arrow heads, Li), and (3) fibrosis = total length of collagen blue stain (dashed black line with in-turned white arrow heads, Lf). Collagen deposition was defined as the length of both loose collagen (light blue with visible background white light) and dense collagen (dark blue) staining (not shown on image). The inflammatory or fibrotic response is calculated as a percent length of the total subcutis (Fig. 5).

Supplementary assessment methods included scoring and capsule measurements. Scoring analysis of the histology images is adapted from the scoring system used by Greenhalgh et al. (1990) [51]. In brief, lower scores for either collagen deposition or inflammation would be given to histology images with minimal or no collagen staining or tissue sections with no immune cell accumulation. Scores closer to 12 would be given to tissue images with highly dense collagen staining or thick layers of inflammatory cells within the subcutis. Scorers were first shown two test images as a means of calibration, and additionally assessed sham control images for method validation (Fig. 5O and Supplemental Fig. 4). A full description of scoring ranges, criteria, and example diagrams is shown in Supplemental Table 1. For images with an implant present at the study endpoint, we also collected measurements of the surrounding layer of inflammatory cells and the fibrous capsule. Inflammatory cells were stained red and identified at the interface of the implant. The collagen-rich fibrous capsule is stained blue and was defined from the interface of the inflammatory cell layer, until either the end of collagen staining or at an observable change in collagen density.

Fibrosis in the fallopian tube is best defined by the by the (1) absence of epithelium and (2) presence of collagen-rich scar tissue in the fallopian tube luminal space. Histology images were therefore visually assessed for these features. To quantify tubal patency in the baboon histology images (Fig. 6), we additionally measured the perimeter of uninterrupted epithelium (yellow dashed line) using ImageJ.

### 2.7 Statistical analysis

Statistical analysis was conducted using GraphPad Prism 8 software. Statistical significance between for fiber diameter measurements, cytokine expression, histology scoring, and measurements was all determined using two-way ANOVA. One-way ANOVA was used to compare cytokine expression of controls and tested fiber/drugs.

## 3 Results and discussion

### 3.1 Design targets and attributes of electrospun fiber formulations to provoke fibrosis

To probe microenvironmental effects on the FBR, we selected polymers commonly employed in drug delivery systems with different timeframes of drug release. PVA and PEO have been used to study factors that impact the development of a fibrous capsule [41], and to formulate solid dosage forms for short-timeframe mucosal drug delivery [52,53]. Polyesters such as PLGA and PCL have been extensively studied as subcutaneous implants due to their biocompatibility, long-term biodegradability, and drug release [54,55]. Using these polymers, we formulated three different blends to elicit known mechanisms in the FBR that culminate in fibrous capsule formation that is characterized by a collagen rich, scar tissue growth at the implant surface. First, water-soluble PVA/PEO fibers, which exhibit rapid dissolution, were used to burst release sclerosants to only induce acute inflammation – the initial phase of the FBR. Second, we used a blend of PLGA and PCL to provide sustained sclerosant delivery and a material depot for inducing persistent and chronic inflammation. Finally, since fibroblasts play a critical role in fibrous capsule formation, PLGA/PCL fiber blends were modified to incorporate gelatin (Gel), which contains RGD-binding sequences shown to enhance fibroblast attachment [56] and macrophage fusion for foreign body giant cell (FBGC) formation [1].

Polymers were formulated with three physicochemically and mechanistically diverse sclerosing agents used to further provoke an immune response and initiate fibrosis (Table 1, Fig. 1). Sclerosing agents were selected from drugs used clinically for fibrosis inducing therapies. Doxycycline (Dox) is a water-soluble tetracycline derivative that has been shown to initiate fibrosis by inhibiting matrix metalloproteinases (MMPs), thereby allowing an overabundance of extracellular matrix (ECM) proteins to deposit [57]. Silver nitrate (SN) salt is a water soluble compound implicated in epithelial cell damage, which contributes to both the acute and chronic inflammatory responses [[58], [59], [60]]. Topically delivered 0.5% SN solution is used clinically to treat burn wounds by promoting tissue healing through stimulating an amplified immune response [61]. Polidocanol (PD) is a non-ionic liquid surfactant that is miscible with water and used intravenously as a 1% PD foam for varicose vein sclerotherapy mediated by endothelial cell lysis [39,62,63]. These distinct agents were screened in our study to best identify an agent appropriate for inducing fibrosis and for probing the intrauterine response.

**Table 1:** Sclerosant fiber dosing for *in vitro* and *in vivo* studies.

Sclerosing Agent	Molecular Weight (g/mol)	Aqueous Solubility/Miscibility (mg/mL)	20% IC50 Cells Free drug dose mg/mL (% loading in fibers formulation)	10% LD50 Mice Free drug dose mg/mL (% loading in fibers formulation)
Dox	1025.89 [88]	50 [88]	0.3 (3%)	2 (20%) [89,90]
SN	169.872 [60]	2450 [60]	0.001 (0.01%)	0.16 (1.6%) [90,91]
PD	494.71 [62]	100 [62]	0.01 (0.1%)	1.6 (16%) [90,92]

*In vitro* toxicity determined experimentally using 3T3 fibroblasts and TZM-bL epithelial cells (Supplementary Fig. 1). *In vivo* doses calculated from reported murine LD50 values.

We successfully electrospun all drugs and polymers into solid dosage forms. The PLGA/PCL/Gel polymer blend had the highest observed drug loading for all sclerosing agents, and Dox showed the highest loading of up to 70 wt% within the polymer compositions (Table 2). The higher loading in PLGA/PCL/Gel is likely a product of greater compatibility of the more hydrophilic drugs with hydrophilic functional groups of gelatin. Similarly in a study of PCL/Gel electrospun fibers by Xue et al. (2014), gelatin improved dispersion of hydrophilic metronidazole at higher drug loadings, specifically as an effect of hydrogen bonding between gelatin's amine and carboxylic acid functional groups with the drug and PCL [56]. The sclerosants SN and PD showed loss of electrospinnability at high loading and could only be formulated at 30–40 wt% in PVA/PEO. This low drug loading is potentially due to high solution conductivity or changes in viscosity, which are factors known to affect Taylor cone formation of the charged polymer solution [45]. Overall, we electrospun sclerosants loaded at relevant doses that could be tested *in vitro* to identify highly immunogenic biomaterials for assessing microenvironment effects *in vivo*.

**Table 2:** Sclerosant fiber properties and release profile.

Fiber Formulation		Fiber diameter (μm)	Observed Max Drug Loading (wt. drug/wt. polymer)	% Drug Encapsulation Efficiency	Time to max release
PLGA/PCL	No Drug	1.4 ± 0.47 (****)	–	–	–
	Dox	1.0 ± 0.19 (****, ###)	60%	98 ± 0.54%	7 d
	SN	1.0 ± 0.37 (****, ####)	50%	~56%	10 d
	PD	2.1 ± 2.3	60%	~104%	7 d

PLGA/PCL/Gel	No Drug	0.96 ± 0.22 (****, ####)	–	–	–
	Dox	1.1 ± 0.19 (****, ##)	70%	81 ± 0.17%	10 d
	SN	0.94 ± 0.25 (****, ####)	60%	~100%	10 d
	PD	1.1 ± 0.22 (****, ##)	60%	~97%	3 d

PVA/PEO	No Drug	1.0 ± 0.37 (****, ####)	–	–	–
	Dox	1.1 ± 0.34 (****, ##)	70%	92 ± 7.0%	6 h
	SN	1.14 ± 0.32 (****)	40%	72 ± 3.5%	NA
	PD	0.99 ± 0.22 (****, ####)	30%	NA	NA

Fiber diameters determined using the cell culture dose, with n = 3 samples, n = 3 images, and n = 20 measurements. Representative fiber images are shown in Supplementary Fig. 2. Encapsulation efficiency quantified for mouse implant dose, with n = 3 samples, and n = 3 measurements. Values reported as mean ± standard deviation. If encapsulation efficiency could not be quantified, the maximum percent drug released is reported.

**** = significantly smaller fibers as compared to PLGA/PCL/PD fibers (p < 0.0001).

##, ###, and #### = significantly smaller fibers as compared to blank PLGA/PCL fibers (p < 0.01, p < 0.001, and p < 0.0001, respectively).

### 3.2 Electrospun materials provide rapid or sustained sclerosing agent release profiles

**Fig. 2:**
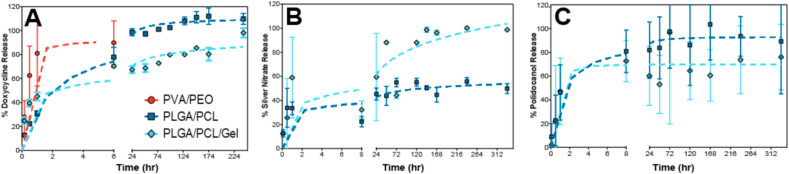
Different fiber formulations vary the release rate of sclerosing agents. Release of (A) Dox, (B) SN, and (C) PD from all fiber blends as percent of total theoretical loading plotted over time from n = 3 fiber sections (mean ± standard deviation).

As expected, PVA/PEO fiber blends showed rapid sclerosant release within an hour, which is consistent with their complete dissolution (Fig. 2, Table 2). Similar studies of PVA fibers have achieved full drug release and material dissolution within 10–30 min [52,53]. In contrast, polyester blends showed biphasic release of sclerosants. Within the first 24 h, an initial burst release from PLGA/PCL fibers accounted for approximately 98% of total loaded Dox, 18% of SN, and 82% of PD. For PLGA/PCL/Gel fibers, the initial burst release accounted for 67% of total Dox, 30% of SN, and 60% of PD (Fig. 2). After 24 h, the second release phase yielded slower release out to a maximum of 10 days with pseudo-linear release rates of approximately 5.8 μg/day PLGA/PCL/Gel/Dox, 1.4 μg/day PLGA/PCL/Gel/SN, and 0.45 μg/day PLGA/PCL/SN) (Fig. 2, Table 2).

We observed that release profiles were dominated by the material composition of the fiber formulations rather than the physicochemical properties of the sclerosants. That is, physicochemically disparate sclerosants like Dox and PD showed similar release from fibers with the same polymer composition like PLGA/PCL (Table 2, Fig. 2). While specific sclerosants such as SN showed some variability in the initial burst release phase from the polyester blends, the cumulative time to 100% release was ultimately the same as the other drugs. These findings are consistent with a study from Carson et al. (2016), which determined the ratio of PLGA/PCL in electrospun fibers and hydrogen bonding between hydrophilic tenofovir and the polyester backbone mediated the controlled release of some drugs beyond 10 days [44]. Overall, we successfully developed polymer blends that achieve either rapid (≤24 h) or sustained agent release (≥7 days) and material persistence. We think these materials are suitable for promoting the FBR since the acute inflammation phase occurs on the order of hours to days, whereas responses that progress to the chronic inflammatory phase occurs over multiple weeks [1]. Based on this understanding, we hypothesize that PLGA/PCL materials that release agent for 10-days will extend into the chronic inflammation phase. In contrast, the PVA/PEO materials would dissolve within hours following implantation and are expected to provide stimuli during the timeframe of the acute inflammation phase.

### 3.3 Polyester electrospun materials induce FBR relevant *in vitro* macrophage cytokine expression

Next, we sought to characterize the immune response generated to our different sclerosant formulations. Activated macrophages have been described to exist in classically (M1) or alternatively (M2) polarized states, both of which are known to influence development of the FBR [64]. M1 macrophages are necessary for the destruction of pathogens, clearing cellular debris, and recruiting immune cells during the initial acute inflammatory phase and results in tissue damage. M2 macrophages attenuate inflammation, but also promote tissue remodeling and the synthesis of ECM proteins such as collagen [[1], [2], [3]]. These two activation modes are phenotypically differentiated by the expression of specific cytokines [[1], [2], [3],^65^]. Moreover, paracrine signaling of cytokines secreted from macrophages during the FBR shapes the response from additional cell types, including other immune cells and fibroblasts [1,3]. Here, we measured the cytokine expression from the murine macrophage cell line RAW 264.7 in response to our materials [25,66]. The cytokines tumor necrosis factor alpha (TNF-α) and interleukin 1 beta (IL-1β) were identified as markers of a pro-inflammatory response characteristic of M1 macrophages. Interleukin 10 (IL-10) expression was quantified as a marker of M2 macrophages [3]. Jones et al. (2007) has shown that biomaterial adherent macrophages are not at first fully classically activated but rather undergo a phenotypic change towards the alternative activation state characterized by secretion of higher concentrations of IL-10 over time [65]. We hypothesized that the FBR would require cytokine expression from both M1 and M2 activated macrophages, including TNF-α, IL-1β, and IL-10 [[1], [2], [3],^64^,^67^,^68^].

**Fig. 3:**
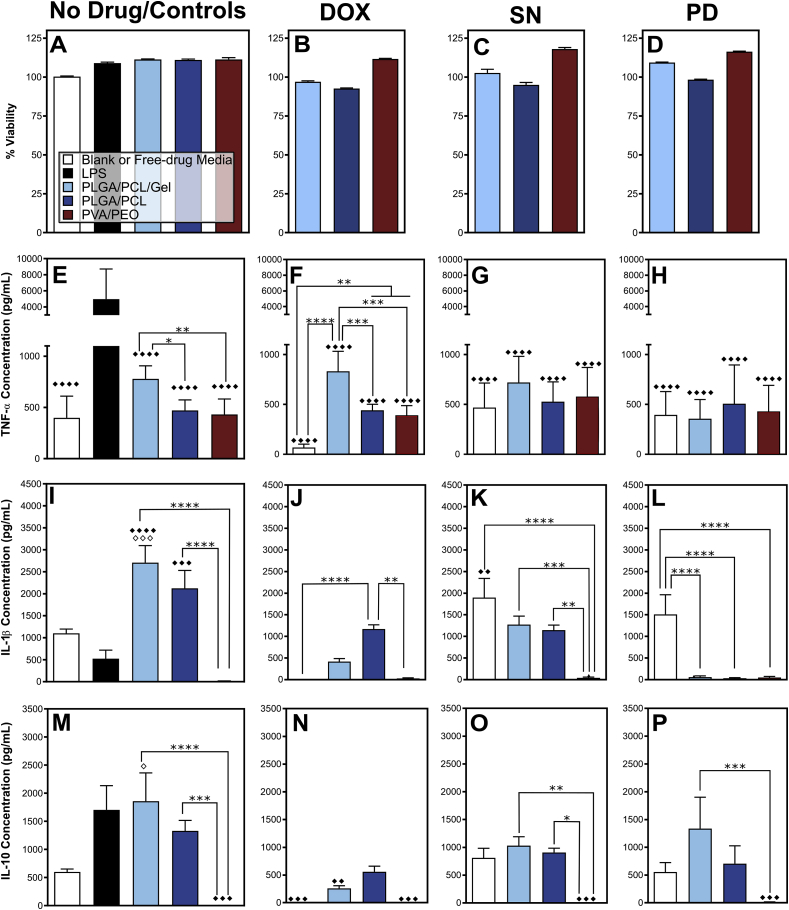
Fibers/drug formulations affect the expression of pro- and anti-inflammatory cytokines by macrophages. (A–D) Cell viability and expression of proinflammatory cytokines (E–H) TNF-α,(I–L) IL-1β, and anti-inflammatory cytokine (M − P) IL-10 from RAW 264.7 macrophages 48-h post-treatment (mean + standard deviation). Viability results are measured for n = 1 cultured fiber sections and n = 3 assay replicates as a percent signal against untreated cells. Cytokine results are measured from n = 3 fiber sections, n = 3 assay replicates. Statistically significant different expression than media (◇) and LPS (◆) is marked on all plots. Statistical differences between material types (*) are specifically marked. For each symbol, * = p ≤ 0.05, ** = p ≤ 0.01, *** = p ≤ 0.001, and **** = p ≤ 0.0001.

For cell culture experiments, we decreased the drug loading within the fibers to be 0.01–3 wt%, or 20% LD50 for each drug, to sustain greater than 90% cell viability (Table 1, Supplementary Fig. 1, Fig. 3A–D). Cells treated with LPS as a positive control expressed the predicted excess of measured cytokines, thereby validating that the cell assay could sufficiently capture the macrophage response. We found that all PVA/PEO fiber formulations induced TNF-α but not IL-1β or IL-10 (Fig. 3). For both SN and PD, IL-1β and IL-10 expression were significantly lower when delivered by PVA/PEO, even when compared to free drug (Fig. 3 K, L, O, & P). A minimal cytokine profile from the PVA/PEO fibers is consistent with our hypothesis that the rapid dissolution of the fibers can capture the early, pro-inflammatory phases of the FBR, but would not sustain a cytokine profile for excessive scar tissue growth or fibrous capsule formation.

Blank PLGA/PCL/Gel and PLGA/PCL/Gel/Dox fibers provoked higher TNF-α expression than other polymer blends (Fig. 3 E & F). Additionally, blank PLGA/PCL/Gel fibers had significantly higher expression of IL-1β as compared to all control groups, and significantly higher IL-10 expression than the media control (Fig. 3 I & M). This suggests that PLGA/PCL/Gel fibers alone could induce the initial inflammatory response leading to fibrotic capsule formation. These data support our hypothesis that material persistence could mediate large immune stimulation. Although these data support a drug-free implant, this study *in vitro* quantified the response from a single cell type at a single timepoint. Therefore, long-term *in vivo* studies of the materials were conducted to further assess the value of sclerosing agent enhanced implants.

### 3.4 Gelatin supplemented polyester implants promote *in vitro* fibroblast attachment

We next assessed *in vitro* attachment of NIH-3T3 fibroblasts to our study materials as a predictive factor in the progression of the FBR *in vivo*. Following the chronic inflammatory phase of the FBR, fibroblasts migrate to the implant surface and generate collagen-rich scar tissue growth to form a fibrous capsule. Fibroblast adhesion and proliferation onto a material surface can therefore inform the capability for implant-tissue integration [56,69]. Based on the cytokine data and slow material degradation, we hypothesized that the polyester fibers could enable cell attachment and thereby promote fibrosis.

**Fig. 4:**
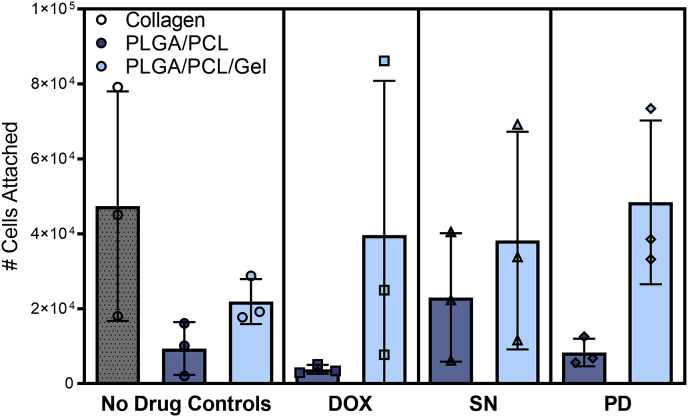
Fiber formulations contribute significantly to fibroblast attachment. Plot of the cell number att atached on the fibers after 24 h as measured by a standard MTS assay. Collagen treated coverslips were used as an attachment positive control. Cell number attached was measured on n = 3 cultured material segments and is plotted as individual measurements with the mean ± standard deviation.

Immediate dissolution of PVA/PEO fibers precluded any ability for cell attachment. Within the polyester blend, we investigated the addition of gelatin due to its (1) properties as a natural polymer with excellent biocompatibility, (2) electrospinnability, (3) RGD binding sequences that may promote cell adhesion, and (4) composition of amine and carboxylic acid functional groups that increase hydrophilicity of the material and has been observed to promote fibroblast attachment and proliferation [1,56,70]. For these reasons, gelatin and synthetic polymer blends are commonly electrospun and studied in tissue engineering applications and for drug delivery [56,70]. However, previous studies also indicate that hydrophilic materials yield less fibrotic encapsulation by decreasing macrophage activation [1,3]. Here, we observe comparable or higher macrophage cytokine expression with our gelatin materials, and stratified analysis of material and sclerosant as factors of attachment indicates that the overall addition of gelatin is a significant factor in mediating fibroblast attachment as determined by 2-way ANOVA (p = 0.0073). Indeed, gelatin blends increased fiber attachment up to four-fold compared to PLGA/PCL fibers. As expected, the sclerosing agents did not significantly affect cell attachment (Fig. 4). Thus, we predict gelatin supplemented materials would generate enhanced fibroblast integration and fibrous capsule formation *in vivo*.

**Fig. 5:**
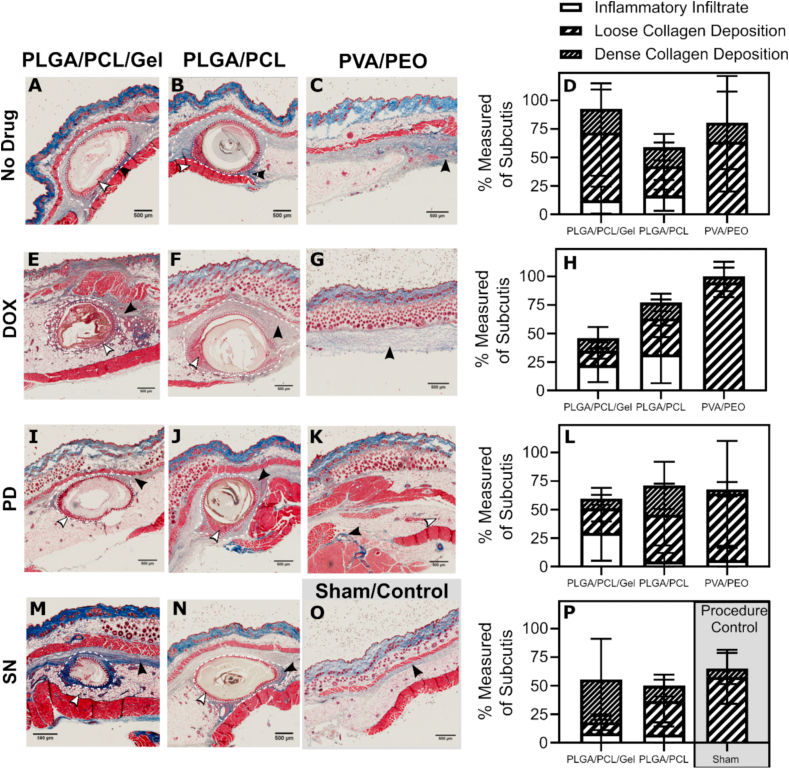
Fiber formulations induce varying degrees of fibrosis: Masson's Trichrome stained tissue sections for (A–C) fiber implants loaded (A–C) without drug, (E–G) doxycycline, (I–K) polidocanol, (M − N) silver nitrate, and (O) the sham/procedure control. Implants are composed of (A, E, I, and L) PLGA/PCL/Gel, (B, F, J, and N) PLGA/PCL, or (C, G, and K) PVA/PEO. Collagen deposition is visualized in blue, cell cytoplasm is stained pink/red. Regions of inflammation (white arrows) and collagen deposition (black arrows) are marked. Images were taken at 2.5× magnification. Quantitative analysis of the histology images is included for fibers with (D) no drug, (H) Dox, (L) PD, and (P) SN/sham. Implant studies and analysis were conducted with n = 1 mouse and n = 2 implant pockets, with the exception of PLGA/PCL/PD and PLGA/PCL/Gel/Dox implants, which were analyzed using two images from a single implant replicate. Percent inflammatory infiltrate and total collagen deposition (loose and dense staining) of the subcutis were determined from representative implant images for each implant (n = 3 sections). Measurements are plotted as mean % ± standard deviation.

**Fig. 6:**
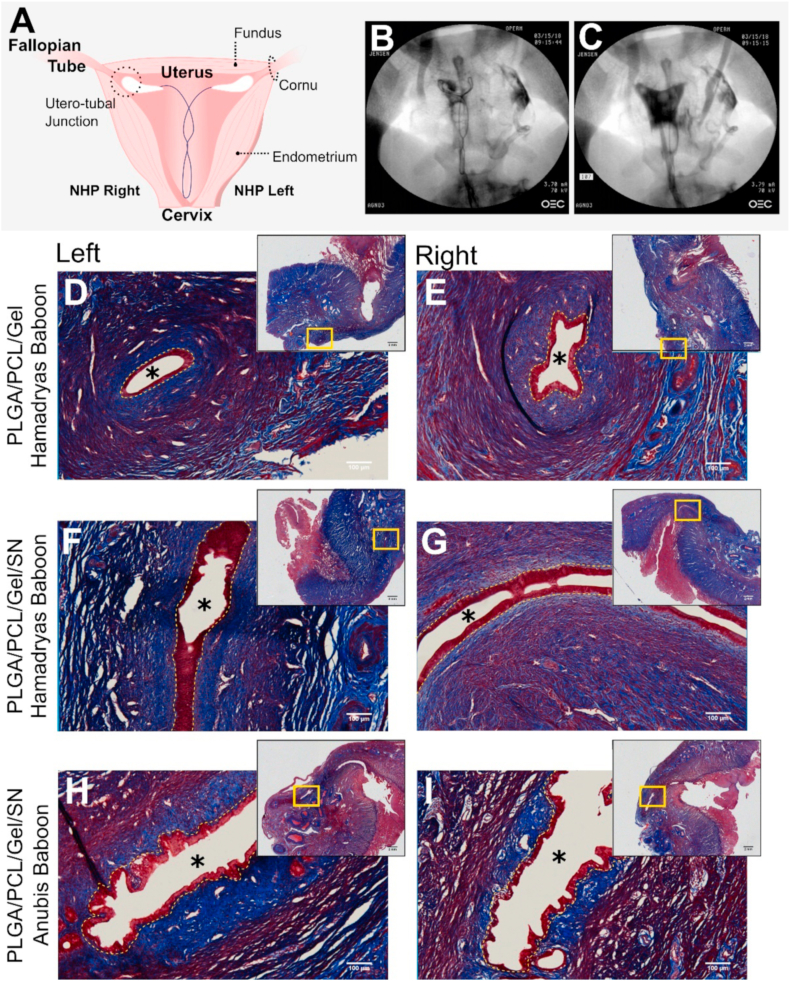
Fibers can be integrated onto IUD devices for intrauterine placement in a non-human primate model. (A) Diagram of the fiber-IUD and relevant anatomical features of the female reproductive tract. Fluoroscopy images of fiber loaded IUD placed in an anubis baboon with active fibers (B) without and (C) with contrast to outline shape of uterine cavity. Resulting tissue sections of left and right cornu of the uterus stained with Masson's trichrome 28-days following treatment with (D&E) PLGA/PCL/Gel fibers without drug in an hamadryas baboon (n = 1), (F & G) 60% (w/w) SN loaded fibers in the anubis baboon (n = 1), and (H&I) SN loaded fibers treated in the hamadryas baboon (n = 1). Main images are captured at 10× magnification, and overview scan images of the tissue sections are included at the top right. Asterisks (*) mark the lumen of the fallopian tube, and yellow-dashed lines outline the tubal epithelium.

### 3.5 PLGA/PCL/Gel/SN implants yield a strong fibrotic FBR within the mouse subcutis

Subcutaneous murine implantation is the standard for assessing the FBR [[25], [26], [27],^42^,^71^], and was used here as a primary outcome to identify study materials that could induce fibrous capsule formation prior to placement in the immune privileged uterine cavity. We assessed subcutaneous histology for the presence of inflammation and collagen deposition. For this, collagen was differentiated between loose and dense deposition, the latter being more indicative of a fibrous capsule-type response [1,42]. End-point tissue response to our study materials were obtained 28-days after biomaterial implantation by taking measurements from representative histology images that captured a large cross-section of the implant (Fig. 5, Supplementary Fig. 3, Supplementary Fig. 4). We performed quantitative image analysis of the histology to rigorously define the fibrotic response to our study materials (Supplementary Fig. 5). As a secondary metric, histology was additionally interpreted via a scoring system (Supplementary Fig. 3 A-L, Supplementary Table 1). Both the quantitative image analysis and qualitative scoring were validated on histology images from our control groups (Fig. 5O and Supplementary Fig. 4), and found to have good congruency (Supplementary Fig. 3).

As expected, the PVA/PEO fibers showed no indication of the implant in the pocket subcutis at the time of necropsy, indicating that they had completely dissolved. Capsule size was not measured for PVA/PEO implants due to the absence of an implant surface boundary (Supplementary Fig. 3 I-O). Inflammation towards PVA/PEO blends was largely undetected as measured by the minimal cellular staining in the adipose tissue, which at homeostasis is comprised of a loose extracellular matrix and dispersed immune cells. Loose collagen deposition was dominantly observed from PVA/PEO treated subcutis histology, with blank and Dox loaded PVA/PEO fibers resulting in quantitatively greater collagen deposition than the sham control (Fig. 5 D, H, & P). As anticipated, PVA/PEO implants did partially initiate the FBR cascade, but comparably less than the polyester implants (Fig. 5C, G, & K).

All polyester-based implants resulted in detectable inflammatory infiltrate of <25% of the subcutis (Fig. 5 D, H, L, & P), which is greater than the sham control (Fig. 5O). We observed that gelatin containing implants (PLGA/PCL/Gel) degraded more than PLGA/PCL implants (Fig. 5A and B, E-F, I-J, & M−N). Bulk degradation is desirable as it would increase implant porosity, which is a feature reported to improve healing responses and promoting greater M2 versus M1 macrophage adhesion [72]. Additionally, RGD-binding sequences present in gelatin are known to promote macrophage fusion, specifically IL-4 mediated macrophages associated with FBGC formation [1,73]. We would therefore expect greater inflammatory cell accumulation around the gelatin supplemented implants as compared to PLGA/PCL alone, which was observed but varied based on the specific combination of implant material and agent. Specifically, PLGA/PCL/Gel/PD and PLGA/PCL/Dox had the largest measurements of immune cell infiltration across the subcutis and surrounding the implant, as compared to the other material types (Fig. 5H & L, Supplementary Fig. 3 N & O). All polyester implants also yielded a detectable fibrous capsule (Fig. 5). The PLGA/PCL/Gel/SN implant resulted in the highest quantity of dense collagen and a clearly defined fibrous capsule (Fig. 5 M & P). We hypothesize that the pro-inflammatory stimuli from SN and pro-healing factors from gelatin contribute to the strong fibrotic response within the subcutaneous microenvironment. Based on this strong fibrotic response, the PLGA/PCL/Gel/SN combination was selected to probe the intrauterine fibrotic FBR.

### 3.6 Intrauterine placement of biomaterials in NHPs yields negligible FBR

To probe the FBR in the uterine cavity, we designed an intrauterine device (IUD) composed of a nitinol wire frame integrated with our reactive biomaterials (Fig. 1C). Smaller animal models, such as rodents, have anatomically different reproductive tracts making intrauterine biomaterial placement difficult or impossible. We specifically selected baboons in this study due to anatomical similarities between the human and baboon cervix, which allows for transcervical placement of materials, and the presence of the intramural portion of the fallopian tube, which is absent in other NHPs [38,40,74]. In transitioning to the larger weight NHP animal model, the total sclerosing agent dosed by our device was significantly below estimated toxic levels for all drugs. Therefore, the potency of silver nitrate and the high maximum 60% (wt./wt.) loading in the PLGA/PCL/Gel blend further supported the use of the PLGA/PCL/Gel/SN materials (Supplementary Fig. 1, Table 2).

One control and two silver nitrate loaded fiber devices were placed in three different baboons for our study to probe the intrauterine fibrotic response. The “fundus-seeking” property of the nitinol IUD consistently places the arms of the frame at the utero-tubal junction, where the fallopian tube diameter is thinnest and most susceptible to fibrosis and occlusion [36,75]. Fibers were therefore wrapped onto each arm of the IUD to target materials to the utero-tubal junction. The materials added to the device could not exceed 1.5 mm thickness in order to accommodate the 3 mm diameter of the transcervical insertion tube, corresponding to 10 mg of fibers per arm (Fig. 1D & E). Initial tests of IUD placement showed that the human-scale nitinol wire frames placed the arms as expected within the utero-tubal junctions in both *Papio hamadryas* (hamadryas) and *Papio anubis* (anubis) (Supplementary Fig. 6). Details of the individual studied NHP subjects and fiber-IUD placement observations are included in Table 3. We evaluated the position of the IUD frames following placement using fluoroscopy with contrast (Isovue). In two NHPs (Control and SN-H), both of the IUD arms localized to one of the cornual regions, and in the third (SN-A) the arms deployed correctly (Table 3, Fig. 6 A & B). This suggests that the adherence of the fibers may have prevented full expansion of the device.

**Table 3:** Summary of NHP subjects and study observations.

NHP	Baboon Species	Biomaterial Treatment	Device Conformation at Placement	Device Conformation at Necropsy	Measured Tubal Perimeter (mm)
Control	*Papio hamadryas*	2x – 10 mg PLGA/PCL/Gel Fiber	Both arms in right utero-tubal junction	Open, device arms local to each utero-tubal junction	1.94 ± 0.50
SN-H	*Papio hamadryas*	2x – 10 mg PLGA/PCL/Gel +60% SN Fiber	Both arms in left utero-tubal junction	Folded at left utero-tubal junction	5.44 ± 3.91
SN-A	*Papio anubis*	2x – 10 mg PLGA/PCL/Gel +60% SN Fiber	Open, device arms local to each utero-tubal junction	Open, device arms local to each utero-tubal junction	6.98 ± 0.51

Perimeter of tubal epithelium measured taken as n = 3 measurements between n = 2 fallopian tubes on n = 1 animal.

We evaluated histologic sections from each cornual region proximal to the IUD arms and found no evidence of inflammation or collagen deposition in either the endometrium or intramural fallopian tube (Fig. 6D–I). At the 28-day study endpoint, no abnormal immune cell infiltration was observed. The wide-angle overview of the uterine cornual histology shows that the ECM is inherently collagen rich, which is supported by other studies of primate uterine tissue [77,78]. We did not observe any excess collagen deposition in these tissue sections as would be evident by tubal obstruction with scar tissue, as well as by a disruption of the tubal epithelium [78]. Rather, tubal epithelium was clearly present and intact in all histology sections (Fig. 6D–I). Additionally, the perimeter of uninterrupted fallopian tube epithelium was measured to be within a range of 1.16–9.62 mm for all treatment groups (Table 3). Considering human fallopian tubes have been reported to be as small as 0.4 mm in inner diameter [79] and up to 4 mm in diameter [80], an epithelial perimeter within a range of approximately 1.26–12.6 mm would be considered typical for healthy human or baboon tubal tissue. PLGA/PCL/Gel fibers persisted beyond 28-days throughout our study, and as anticipated these materials were present for the entirety of intrauterine placement. The absence of observable scar tissue integration onto the material further indicates that the materials did not undergo fibrotic encapsulation. Despite our observations of subcutaneous fibrotic encapsulation of PLGA/PCL/Gel/SN materials in the mouse, the same materials with an increased sclerosing agent dose did not induce a reaction within the uterine environment.

One significant difference between the subcutaneous and uterine environments here is the procedure used to implant the materials. Blood and biomaterial interactions are implicated in the first stage of the FBR cascade, and blood contact is a necessary first step to fibrous capsule formation. Both the subcutis and tubal submucosa are highly vascularized [81,82], but surgical implantation within the subcutis entails greater physical tissue damage than transcervical delivery induces onto the utero-tubal junction. However, the material coated devices did contact blood caused from tissue damage during cervical dilation. Additionally, subcutaneous sham procedures, healed with negligible fibrosis (Supplementary Fig. 4L). For these reasons, the differences between placement procedures are not expected to entirely account for the differences in the responses observed towards the biomaterials in the different compartments.

The delivered dose in our study had a comparably lower mass of total drug than other intrauterine sclerosing agent studies. However, the dose was expected to be sufficient for local epithelial toxicity due to targeted drug delivery to the utero-tubal junction and higher degree of sclerosant/material tissue contact and residence. We do not think that the duration of dose release in this case to be the cause of the minimal response. We expect that prolonged exposure to silver nitrate could delay development of collagen deposition and remodeling. However, having a larger sclerosant dose could induce a stronger fibrotic response to the treatment. For example, our silver nitrate dose in the baboons was approximately 6 mg per utero-tubal junction and did not lead to a fibrotic FBR in the baboons. Neuwirth et al. (1971) showed that a 10% silver nitrate ointment fully infused in pigtail macaque fallopian tubes induced scar tissue occlusion – a dose calculated to be 18.8 mg for an estimated 2 mm diameter by 6 cm long tube [58]. Knowing that fibrosis is a possible reaction of provoked uterine tissue, yet was not observed here in response to material implants confirmed to be reactive in the subcutis, emphasizes the ability of host factors to strongly dampen the fibrotic response.

Although the presence of uterine immune privilege towards foreign bodies such as paternal cells and fetuses has been well documented [[28], [29], [30]] to the best of our knowledge ours if the first study to directly compare the FBR between the subcutis and the uterine environment and demonstrate a dampened fibrotic response in the uterine microenvironment to known fibrosis provoking implants within the subcutaneous microenvironment. Our results illustrate the extent that uterine immune privilege has towards the tolerance of biomaterial foreign bodies. Previous studies have demonstrated that the FBR does vary in magnitude towards different biomaterial properties [1] and across different tissue implantation sites [9]. While it is true that fibrosis can be induced within the immune privileged uterine tissue environment, we showed that a fibrotic response within the uterus is significantly dampened in comparison to classical microenvironments and may require an acute stimulus to provoke. Examples of stimuli needed beyond the presence of a foreign body may include mechanical properties as is the case of the Essure contraceptive device [83], large and repeated drug dosages for chemical tubal occlusion [36,37,39,58], and chronic inflammation from sources such as sexually transmitted pathogens [35,36].

Our work demonstrates the important role that the tissue microenvironment plays in the FBR. Studies are needed to identify the ques, or combination of ques, responsible for this dampened intrauterine fibrotic response. Once identified, these immunosuppressive factors from the uterine microenvironment could be used in a peripheral biomaterial implant model. Such studies would inform possible methods for translating this immune privilege to implants broadly. For example, both progesterone and estrogen are steroid hormones produced cyclically by the ovaries. Both hormones are known to regulate the quantity of macrophages present within the uterus, as well as the expression of these macrophages in the anti-inflammatory, M2 polarization state [84]. The expression of these hormones, as well as the local immune environment, changes dynamically throughout the menstrual cycle [30,76,85]. Some studies of progesterone and estrogen's effects on the FBR have already been studied [86,87], but the direct effect of both hormones on a subcutaneous implant has not been considered. Additionally, the temporal effects of such hormones could be studied in the context of the FBR. The role of macrophages as an important mediator in uterine immune privilege has already been identified, especially in the context of pregnancy. In the context of the current work, we expect that studies of macrophage polarization, specifically in response to various biomaterials, would further elucidate the presence of the intrauterine FBR dampening response.

## 4 Conclusions

In this study, we designed a novel biomaterial-IUD to investigate the fibrotic FBR within the uterine environment. Electrospun fibers were used for their ability to (1) formulate both hydrophilic or hydrophobic polymers, (2) incorporate ECM-like fiber matrices including natural polymers with RGD-binding sequences, and (3) be easily integrated onto an intrauterine device for direct delivery to the utero-tubal junction. These fibrous materials also showed high encapsulation efficiency of the physicochemically diverse agents Dox, SN, and PD.

Polyester fibers successfully released sclerosing agents up to 10 days. These materials additionally persisted as a fiber depot beyond 28 days. In contrast, PVA/PEO blend fibers fully dissolved and released the drug cargo within an hour as designed to capture only the initial phases of the FBR. In assessing the *in vitro* immune response, polyester blend fibers were shown to have higher pro-inflammatory (IL-1β) and pro-healing (IL-10) cytokine expression compared to than PVA/PEO fibers. This suggests that sustained polyester fiber blends, and even these fibers alone, are more likely to provoke the initial cell signaling needed to cause permanent fibrotic scar tissue growth. Using the subcutaneous mouse implant model, the fibrotic reaction to these materials was also captured, showing high collagen-rich tissue encapsulation of PLGA/PCL/Gel/SN fibers. PLGA/PCL/Gel/SN was prioritized for assessing the fibrotic FBR in the intrauterine NHP model due to the relative potency of SN and the higher SN loading within the gelatin blend fibers.

Histological evidence from the NHP studies indicate that the materials optimized for initiating subcutaneous fibrosis did not cause excess intrauterine fibrotic tissue growth 28-days post placement. This study illustrates the significant role of the immunosuppressive tissue microenvironment on the FBR. Knowledge from this study highlights the importance of considering the tissue environment in designing biomaterials. Future studies could additionally seek out these mechanisms of protection or could investigate methods of bringing known elements of immune privilege to systemic implants.

## Credit author statement

**Jamie L. Hernandez**, Conceptualization, Methodology, Formal analysis, Investigation, Visualization, Writing – original draft. **Jaehyung Park**, Investigation, Writing – review & editing. **Shan Yao**, Investigation, Formal analysis. **Anna K. Blakney**, Conceptualization, Writing – review & editing. **Hienschi V. Nguyen**, Investigation. **Bob H. Katz**, Conceptualization, Resources. **Jeffrey T. Jensen**, Conceptualization, Formal analysis, Funding acquisition, Writing – review & editing. **Kim A. Woodrow**, Conceptualization, Supervision, Formal analysis, Funding acquisition, Writing – review & editing.

## Data statement

The raw and processed data used in this study are available to download from https://doi.org/10.17632/cv793bnw35.2.

## Declaration of competing interest

The authors declare the following financial interests/personal relationships which may be considered as potential competing interests: Dr. Jensen has received payments for consulting from Abbvie, Cooper Surgical, Bayer Healthcare, Evofem, Mayne Pharma, Merck, Sebela, and TherapeuticsMD. OHSU has received research support from 10.13039/100006483Abbvie, Bayer Healthcare, Daré, Estetra SPRL, Medicines360, 10.13039/100004334Merck, and Sebela. These companies and organizations may have a commercial or financial interest in the results of this research and technology. These potential conflicts of interest have been reviewed and managed by OHSU. Bob Katz is a paid consultant for Sebela Pharmaceuticals and CEO of Hybridge Medical, LLC.
