# Supplementary material for: Effect of tissue microenvironment on fibrous capsule formation to biomaterial-coated implants
Source: Biomaterials. 2021 Jun;273:120806. doi: 10.1016/j.biomaterials.2021.120806 (PMC8135119; doi:10.1016/j.biomaterials.2021.120806)

#

# Supplemental Data

**Supplementary Fig. 1:** **Cell cytotoxicity of free drug to determine LD50 in vitro.** Dose-response curves for 3T3 fibroblasts and TZM-bL epithelial cells treated for 24 hours with (A) doxycycline, (B) silver nitrate, and (C) polidocanol (n=3). Viability was measured using a CellTiter Blue assay and calculated based on cell signal from untreated cells. Data is fit with a non-linear, log(agonist) vs response, least squares equation (using GraphPad Prism). LD50 values estimated at the intersection of the data fit at 50% viability.


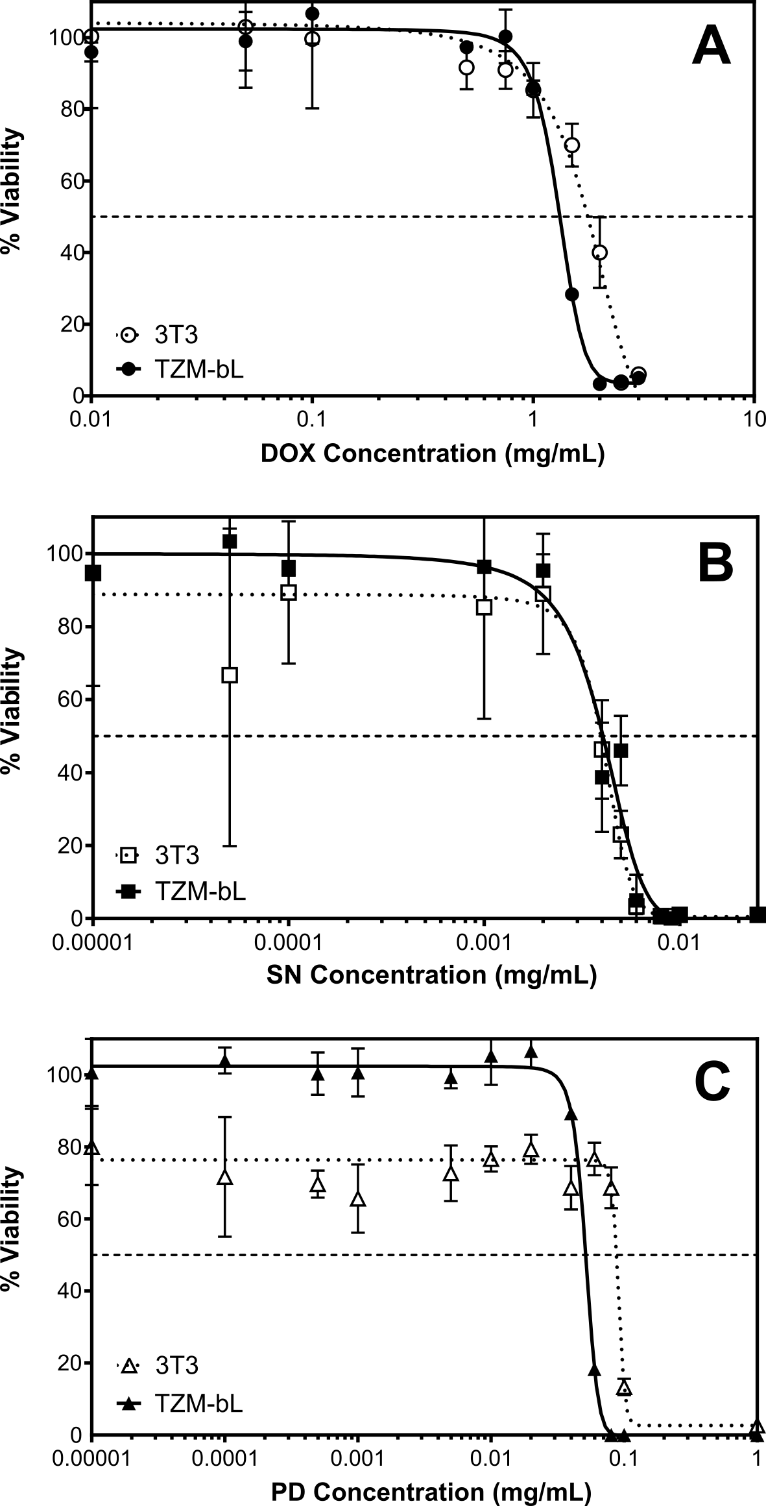


**Supplementary Fig. 2:** **Images of differences in fiber morphology.** Bright field images of the different fiber/drug combinations electrospun onto microscope slide coverslips captured at 40x magnification by a brightfield microscope (Nikon Eclipse Ti).


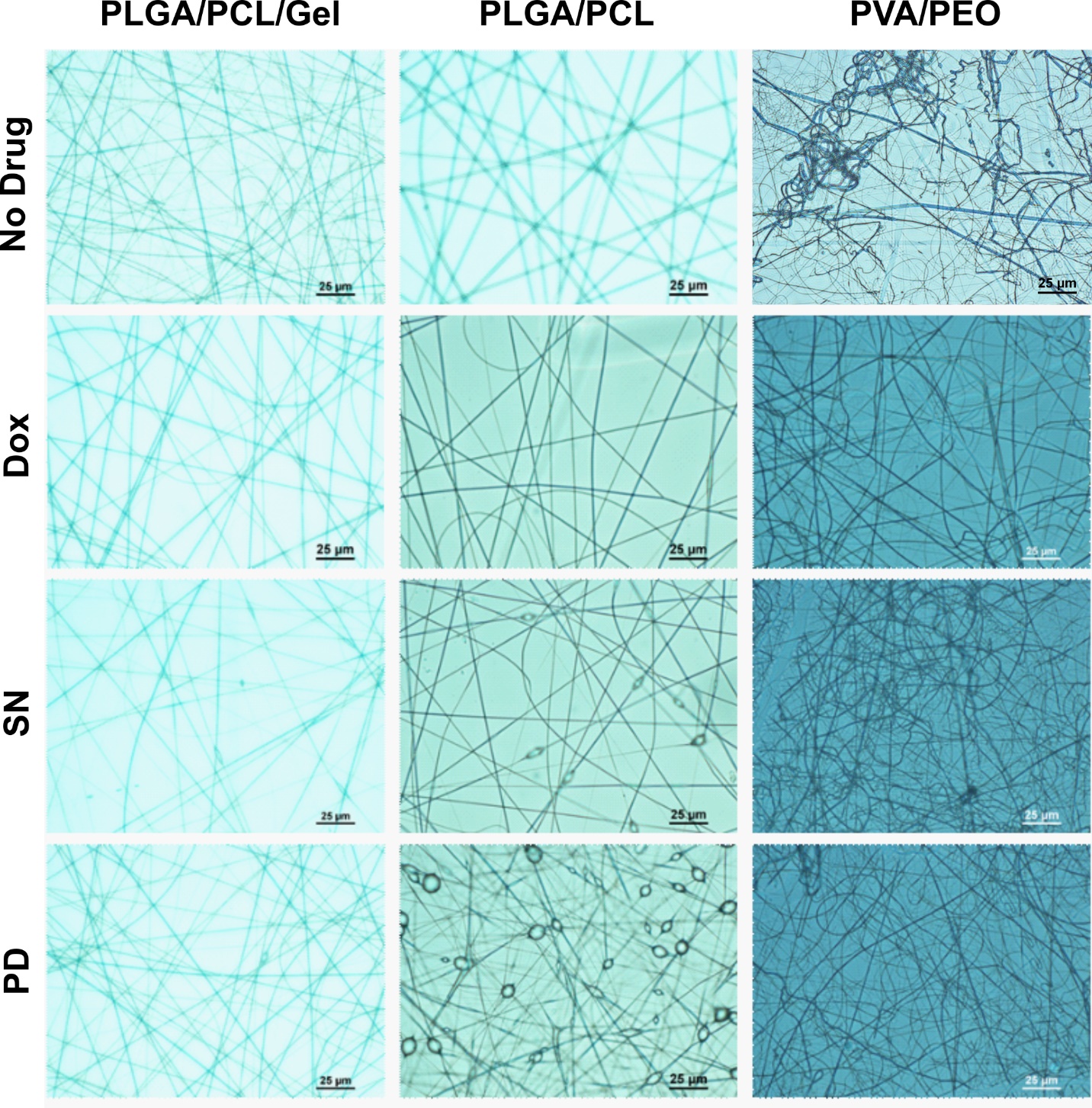


| **Supplementary Table 1:** Table of descriptions and diagrams used to score histology images | | |
| --- | --- | --- |
| **Criteria: Collagen Deposition** | | |
| **Score** | **Description** | **Diagram Example** |
| 1-3 | Minimal collagen deposition. Loose deposition if at all. | 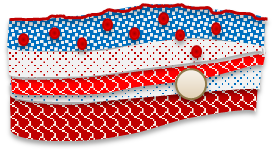 |
| 4-6 | Thin layer of collagen deposition. Deposition of collagen is loose between muscle layers. | 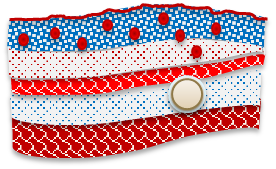 |
| 7-9 | Thick collagen deposition around implant or between muscle layers. Some regions of thick deposition, but primarily loosely oriented. | 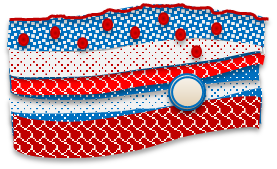 |
| 10-12 | Extensive and dense collection of collagen found around implant or between muscle layers. | 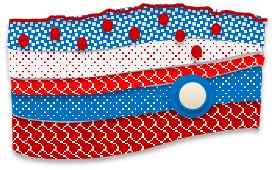 |
| **Criteria: Inflammatory Infiltrate** | | |
| **Score** | **Description** | **Diagram Example** |
| 1-3 | No Apparent accumulation of inflammatory cells. | 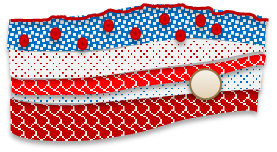 |
| 4-6 | Few inflammatory cells are present in the pocket space or in adipose tissue. | 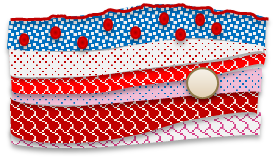 |
| 7-9 | A thin layer of cells surrounds the implant or are accumulated in the pocket space. | 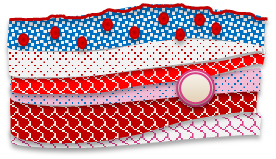 |
| 10-12 | A thick layer of cells surrounds the implant or are accumulated in the pocket space. | 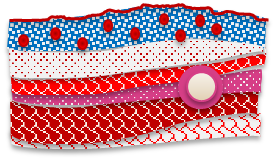 |

**Supplementary Fig. 3:** **Scores,** **Fibrous capsule, and cell layer measurements from implant histology**. Implants made of PLGA/PCL/Gel (light blue), PLGA/PCL (dark blude), and PVA/PEO (red) elute (A, E, I, & M) no drug, (B, F, J, & N) doxycycline, (C, G, K, & O) polidocanol, or (D, H, L, & P) silver nitrate. Scores for collagen deposition (A-D) and inflammation (E-H) were determined by the scoring system shown in supplementary Table 1. Scores were determined by n=8 scorers, and n=2 images of implants for n=1 mouse. Measurements of (I-L) fibrous capsule and inflammatory cell layers (M-P) taken from Masson’s Trichrome histology images at the implant site. Biomaterial implants included in this measurement analysis were present at the time of sacrifice, and therefore are composed of PLGA/PCL or PLGA/PCL/Gel. Capsule quantifications were made using ImageJ and n=8 measurements of n=2 implants. Implants for PLGA/PCL/PD and PLGA/PCL/Gel/Dox were only imaged for as n=1 implant. Measurements are plotted as individual measurements as well as bars that represent the average ± the standard deviation.


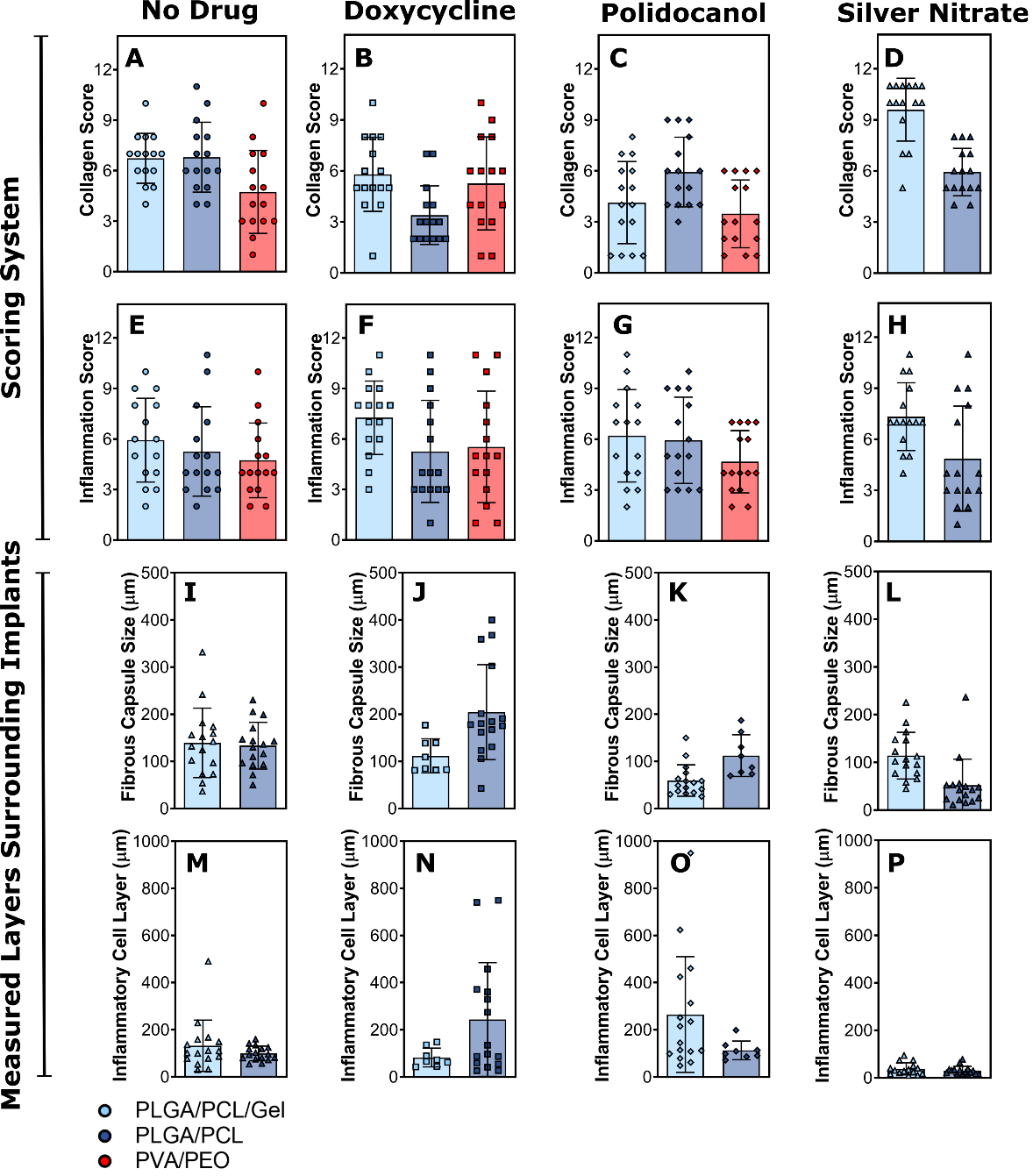


**Supplementary Fig. 4: Murine implant internal control histology sections**. Masson’s Trichrome stained tissue sections sampled from implant pocket site, away from the location of the implants. Biomaterial implants are composed of (A, D, G, & J) PLGA/PCL/Gel, (B, E, H, & K) PLGA/PCL, and (C, F, & I) PVA/PEO and eluted (A-C) no drug, (D-F) Dox, (G-I) PD, or (J-K) SN. Histology taken from a (L) sham procedure control is additionally included. Images captured as scans using a Sakura VisionTek Digital Microscope.

--
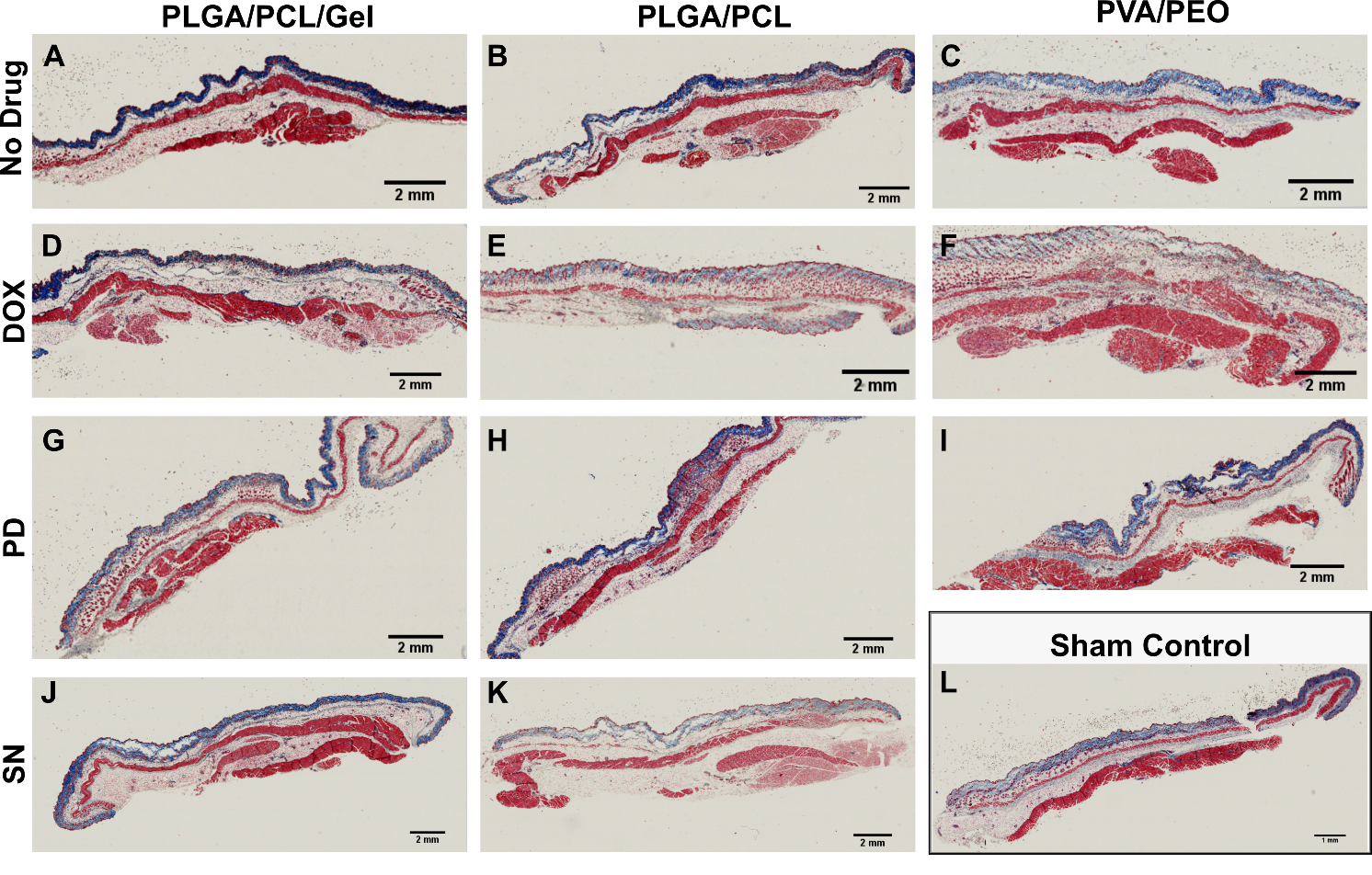

**Supplementary Fig. 5. Diagram of histology quantification method.** Masson’s Trichrome stained PLGA/PCL/Gel no drug implant tissue is shown as an example for the quantification method. Three regions of interest (ROI) were defined to perform unbiased measurements. The first ROI is defined by a line, intersecting the center point of the implant in the cross-section image, and drawn perpendicular to the skin surface and through the lower layers of muscle (L1, solid white line). Two additional ROIs are defined as parallel lines (solid white line) 1 mm to the left (L2) and right (L3) of L1. ImageJ (FIJI/ImageJ 1.51s, National Institutes of Health, USA)^50^ was used to take three measurements at each of these ROIs: (1) subcutis length = length between the two muscle layers (dashed white line, Lsc), (2) inflammation = total length of accumulated immune cells stained red within the subcutis, and additionally identified by cell structure (dashed black line with in-turned black arrow heads, Li), and (3) fibrosis = total length of collagen blue stain (dashed black line with in-turned white arrow heads, Lf). Collagen deposition was defined as the length of both loose collagen (light blue) and dense collagen (dark blue) staining (not shown on image). The inflammatory or fibrotic response is calculated as a percent length of the total subcutis. The inflammatory or fibrotic response is calculated as a percent length of the total subcutis


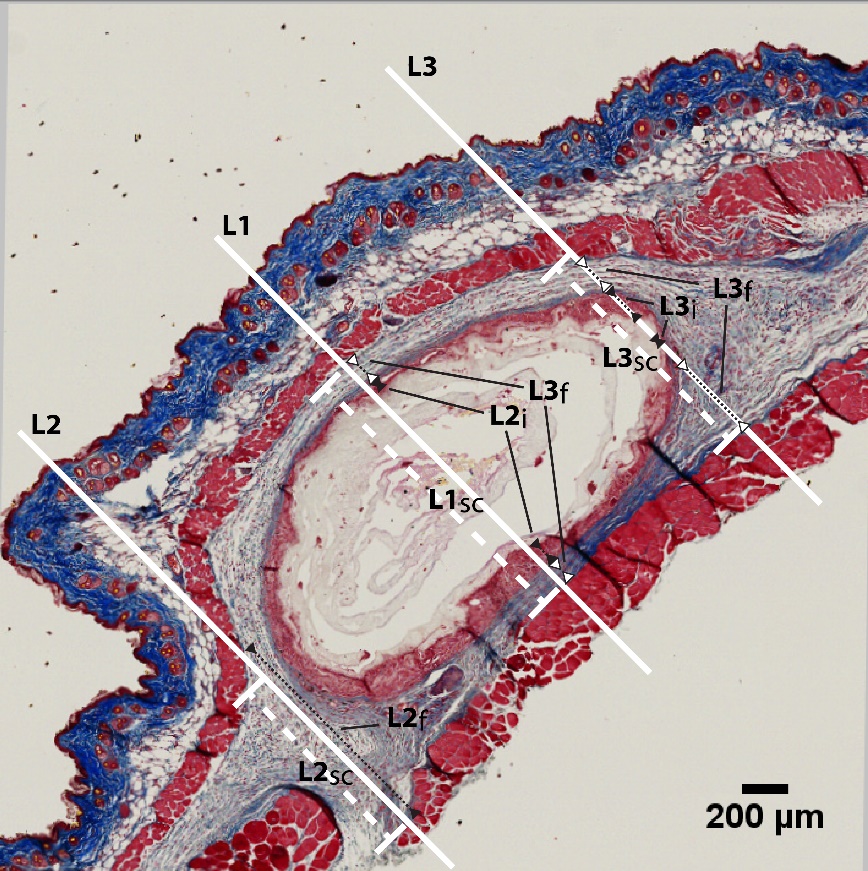


**Supplementary Fig. 6:** **X-ray images of ContraMed VeraCept IUDs placed in hemadryas (A & B) and Anubis (C) baboons**. The space of the uterine cavity was imaged using contrast agent.


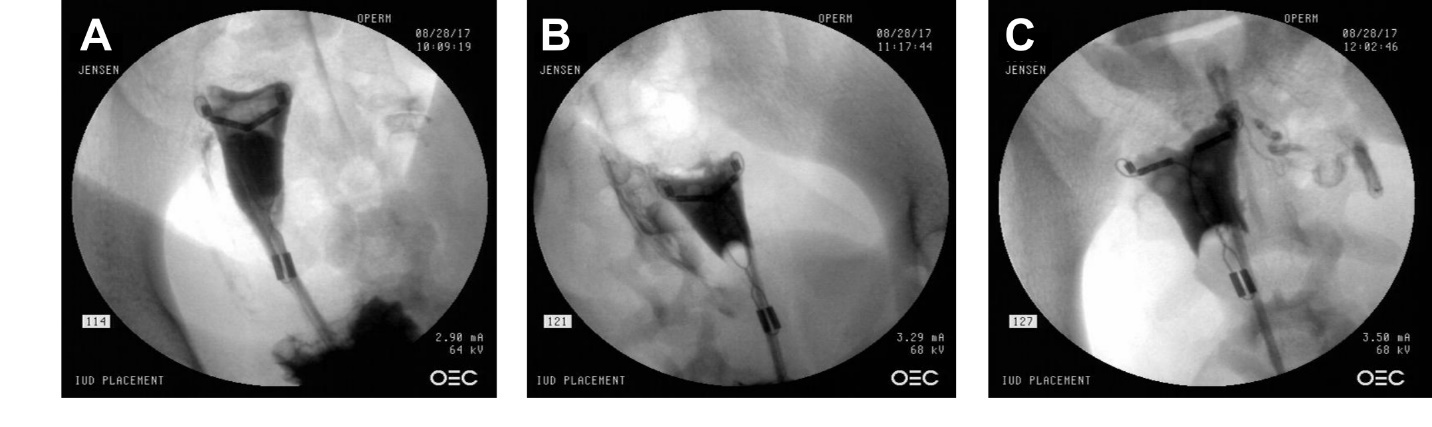

Supplement: Multimedia component 2 [file mmc2.docx]
